# Health Service Leaders’ Perspectives on Type 1 Diabetes Models of Care for Children and Young Adults in Australia: A Mixed‐Methods Study

**DOI:** 10.1155/jdr/7441677

**Published:** 2026-04-29

**Authors:** Yvonne Zurynski, Carolynn L. Smith, Ann Carrigan, Nehal Singh, Timothy W. Jones, Leanne Cromb, Helen J. Woodhead, Anthony Pease, Tony Huynh, Ann M. Maguire, Kristen A. Neville, David E. Bloom, Sophia Zoungas, Jenny Couper, Jeffrey Braithwaite, Elizabeth A. Davis

**Affiliations:** ^1^ Centre for Healthcare Resilience and Implementation Science, Australian Institute of Health Innovation, Macquarie University, Sydney, New South Wales, Australia, mq.edu.au; ^2^ Faculty of Medicine and Health, The University of Sydney, Camperdown, New South Wales, Australia, sydney.edu.au; ^3^ Children’s Diabetes Centre, The Kids Research Institute Australia, University of Western Australia, Perth, Western Australia, Australia, uwa.edu.au; ^4^ The Sydney Children’s Hospital Network, Westmead, New South Wales, Australia, schn.health.nsw.gov.au; ^5^ The University of New South Wales, Sydney, New South Wales, Australia, unsw.edu.au; ^6^ School of Public Health and Preventive Medicine, Monash University, Melbourne, Victoria, Australia, monash.ac.za; ^7^ Children’s Health, Queensland Clinical Unit and Faculty of Medicine, The University of Queensland, Brisbane, Queensland, Australia, uq.edu.au; ^8^ Institute of Endocrinology and Diabetes, The Children’s Hospital at Westmead, Westmead, New South Wales, Australia, nsw.gov.au; ^9^ The Children’s Hospital at Westmead Clinical School, Faculty of Medicine and Health, The University of Sydney, Camperdown, New South Wales, Australia, sydney.edu.au; ^10^ Department of Endocrinology, Sydney Children’s Hospital Randwick, School of Clinical Medicine, University of New South Wales, Sydney, New South Wales, Australia, unsw.edu.au; ^11^ Harvard University, Boston, Massachusetts, USA, harvard.edu; ^12^ Department of Endocrinology and Diabetes, Alfred Health, Melbourne, Victoria, Australia, alfred.org.au; ^13^ The Women’s and Children’s Hospital, North Adelaide, South Australia, Australia, wch.sa.gov.au; ^14^ University of Adelaide, Adelaide, South Australia, Australia, adelaide.edu.au; ^15^ Department of Endocrinology and Diabetes, Perth Children’s Hospital, Nedlands, Western Australia, Australia

## Abstract

**Introduction:**

Type 1 diabetes (T1D) is a lifelong condition typically diagnosed in childhood. Clinical practice guidelines recommend comprehensive multidisciplinary team (MDT)‐based care led by paediatric endocrinologists. However, experiences and opinions of health professionals about the implementation of T1D MDTs in Australia are currently unknown.

**Aims:**

To describe health service teams caring for children and youth with T1D in Australia and to identify opportunities for service improvements from providers’ perspectives.

**Methods:**

Mixed‐methods study co‐designed with clinicians and consumers, including a survey of clinic leaders and semi‐structured interviews. Survey questions covered modes of care delivery, team composition and outreach. Interview transcripts were thematically analysed using a hybrid inductive/deductive approach.

**Results:**

Thirty‐two T1D services leaders completed the survey; 16 were from major cities and 16 were from regional/rural areas across all Australian states and territories. The services provided care for ~51% of all <19‐year‐olds living with T1D. T1D services were multidisciplinary and commonly included dieticians (*n* = 29, 94%), nurse diabetes educators (*n* = 22, 71%) and general paediatricians (*n* = 21, 68%). Eight (29%) services had a dedicated psychologist. A quarter (25%) of regional/rural services had a paediatric endocrinologist compared with 100% of major city services (*χ* ^2^ = 18.355; *p* < 0.001). All services offered telehealth consultations. Interviews revealed that services placed high value on having established cohesive teams skilled in T1D. Service leaders had concerns regarding workforce capacity and shortages, limited access to psychologists, inequitable access to insulin pumps and limited links with general practitioners.

**Conclusion:**

This mixed‐methods study is the first Australia‐wide exploration of T1D models of care that describes care provision from the clinicians’ perspectives. A need exists to address current gaps to achieve the recommended MDT models of care for T1D. Understanding existing models of care will be essential to determine the future impacts of changes in policies, therapies and demands on paediatric T1D services.

## 1. Introduction

Type 1 diabetes (T1D) is a chronic condition that is often diagnosed in childhood [1]. In 2021, an estimated 13,200 people under the age of 19 years were living with T1D in Australia [2]. Despite the development of evidence‐based clinical guidelines for T1D management and availability of new technologies, such as continuous glucose monitors (CGMs), insulin pumps and hybrid closed‐loop therapy [3], children living with T1D still have a shorter life expectancy by ~12–16 years compared with children without T1D [4]. This disparity can in part be attributed to poor glycaemic control, which can lead to complications, such as cardiovascular disease [5], diabetic kidney disease, neuropathy and retinopathy [6]. Concerningly, 85% of children and young adults living with T1D struggle to achieve and maintain glycaemic control [7, 8]. Given the prevalence of, and the dire health consequences arising from, suboptimal glycaemic control, optimal care management and support must be provided to children and youth with T1D and their families to improve outcomes [9].

To support optimal care management, the International Society for Pediatric and Adolescent Diabetes (ISPAD) publishes and regularly updates Clinical Practice Consensus Guidelines [10] for T1D management [10]. Central to the ISPAD guidelines are recommendations on the provision of comprehensive healthcare and support through multidisciplinary teams (MDTs) comprising specialist doctors (paediatricians, paediatric endocrinologists), specialist nurses, diabetes educators, social workers and psychologists [9]. Care provided by a skilled MDT can improve patient outcomes and provide continuity of care and support for patients and families to manage T1D more effectively [11, 12]. Another key recommendation is for specialist T1D MDTs to link up with primary care providers, including general practitioners, to ensure care continuity.

The extent to which the recommended MDT approach has been implemented is currently unknown, and the composition of existing MDTs in Australia has rarely been described [12]. Understanding the current structures and compositions of teams and gaining service providers’ perspectives on how current T1D services are functioning and the barriers and enablers they experience are important for continual improvement of service delivery.

This study aims to understand the current models of care available for the provision of T1D services for children and youth living in Australia, focusing on the following model components:

• Multidisciplinary teamwork and team composition.
• Links with local primary healthcare networks and primary care services.
• Psychological and other types of nonmedical support provided (e.g., referral to peer support groups, apps, liaising with schools).
• The use of telehealth consultations where appropriate.

The study also sought to describe experiences and opinions of leading health professionals working in T1D services about aspects of their models of care that are working well and priorities for improvement.

## 2. Methods

The study followed the previously published protocol, which described the research methods and approach in detail [13]. The following briefly describes the methods. No artificial intelligence generated content (AIGC) tools, such as ChatGPT, nor others based on large language models (LLMs) were used in developing any portion of this manuscript.

### 2.1. Study Design and Setting

A mixed‐methods study was designed and executed using a comprehensive online survey of T1D service providers in addition to undertaking qualitative interviews with service leaders. Services located in metropolitan, regional and rural areas across all states and territories of Australia were invited to participate. In Australia, multidisciplinary specialist care for T1D is mostly provided by paediatric T1D clinics based in the eight children’s hospitals across Australia [14]. In jurisdictions where there are no stand‐alone children’s hospitals (e.g., in the Northern Territory, Australian Capital Territory and in Tasmania and in regional areas) paediatric T1D clinics may operate in general hospitals [14].

### 2.2. Recruitment

Healthcare leaders who provide care for children and youth living with T1D were recruited for the survey through the Australian and New Zealand Society for Paediatric Endocrinology and Diabetes (formerly the Australasian Paediatric Endocrine Group [APEG]), the Juvenile Diabetes Research Foundation’s Global Centre of Excellence, local clinicians’ networks, newsletters and through snowballing. We requested that the most senior provider working in each service complete the survey or nominate an appropriate proxy.

### 2.3. Data Collection

#### 2.3.1. Survey of T1D Services

Using a survey previously developed in the United Kingdom as an initial guide [15], a working group—comprising paediatric endocrinologists (*n* = 4), a clinical diabetes educator, a health consumer and health services researchers (*n* = 3)—convened to co‐design the survey questions and ensure their relevance for the Australian setting. Ninety‐nine survey questions were grouped across 20 topics, including responder demographics (profession, age group, gender, postcode of clinic); clinic setting (hospital outpatients, private rooms); team members and their professions and care delivery modalities (face‐to‐face, telehealth). The survey questions included various response options, such as direct numerical responses, Likert scales, check lists and short text answers. The anonymous survey was developed and administered via REDCap [16] (see Supporting Information 1: Supplemental File 1). The final survey question gave respondents the opportunity to participate in a follow‐up qualitative interview by providing their email address, which was stored separately from the questionnaire data to maintain the anonymity of the survey responses. Data collection occurred from April to July 2022.

#### 2.3.2. Qualitative Interviews

Respondents who provided their contact details were contacted to arrange a time for an interview. All participants received an information sheet and consent form. Participants signed the consent form, and consent was verbally confirmed at the beginning of the interview. Interviews were completed by one of the research team (either Ann Carrigan or Nehal Singh) either in person or on Microsoft Teams. Participants could opt in to have the conversation recorded, and four of the seven interviews were audio‐recorded. Meticulous notes were also taken during interviews to ensure accuracy. Recorded interviews were auto transcribed via Microsoft Teams, and transcripts were checked for accuracy against the recordings and corrected. Corrections included changing misspelled words and minor grammatical changes that did not alter the meaning of the transcript. The transcripts were de‐identified by assigning a code number.

Participants were asked the following questions with the interviewer exploring issues raised by the participants and using conversational prompts when needed:

• What is working well in your service?
• What is not working well in your service?
• Do you have anything else you would like to add?

### 2.4. Data Analysis

Quantitative and qualitative data were collected sequentially and analysed separately.

The survey data were analysed in SPSS (Version 26) using descriptive statistics, chi‐square tests and within‐subjects statistical tests for paired samples (McNemar test) as appropriate, including effect size (Cramér’s V). An effect size ≥0.50 was considered to be strong, 0.30 was moderate, ≤0.10 was small [17]. Missing data were managed through listwise deletion. Service location was determined based on postcode and classified into two categories (major city and regional/remote) using the Australian Standard Geographical Classification System [18]. The free‐text responses to open‐ended questions in the survey were thematically analysed for common themes. Transcripts of the qualitative interviews and metadata were imported into NVivo V.20 software [19]. Data were thematically analysed by two researchers (Ann Carrigan and Nehal Singh) using a hybrid deductive/inductive approach to identify data‐driven emerging patterns using an open coding process [20]. The researchers met regularly to discuss and refine coding and identify broad themes to ensure intercoder reliability. Themes were verified by a senior author (Yvonne Zurynski) and further discussed in meetings with other authors (Elizabeth A. Davis, Timothy W. Jones, Leanne Cromb, and Carolynn L. Smith).

### 2.5. Ethics Approval

This study was performed in accordance with the Declaration of Helsinki and approved by the Faculty of Medicine, Health and Human Sciences Research Committee, Macquarie University (#520221154439676).

### 2.6. Funding

This project was supported by the Breakthrough T1D (#5_SRA‐2021‐1088_M‐X).

## 3. Results

### 3.1. Survey Results

Thirty‐two service providers consented to and completed the online survey. Twenty‐two completed the whole survey, and 10 completed parts of the survey.

#### 3.1.1. Participant Characteristics

Fifteen providers (47%) were aged between 41 and 50 years (31–40 years: *n* = 5 [16%], 51–60 years: *n* = 8 [25%], >61years: *n* = 4 [13%]). Twenty (63%) were female. Sixteen (50%) were paediatric endocrinologists, 14 (44%) were paediatricians interested in T1D and/or endocrinology and two (6%) were specialist nurses or nurse diabetes educators (*n* = 2, 6%).

#### 3.1.2. Service Description

Half of the services were located in major cities (*n* = 16); the other half in regional/rural (*n* = 16) areas of Australia (Table 1) [18].

**Table 1:** Survey responses across Australian states and territories by remoteness and location.

Location				
State/territory	Total number of services, *n* (%)	Major city, *n* (%)	Regional, *n* (%)	Remote, *n* (%)
Queensland	12 (38)	3 (25)	7 (58)	2 (17)
New South Wales	9 (28)	6 (67)	3 (33)	—
Tasmania	3 (9)	1 (33)	2 (67)	—
South Australia	3 (9)	3 (100)	—	—
Victoria	2 (6)	—	2 (100)	—
Western Australia^a^	1 (3)	1 (100)	—	—
Northern Territory^a^	1 (3)	1 (100)	—	—
Australian Capital Territory^a^	1 (3)	1 (100)	—	—
Total	32	16 (50)	14 (44)	2 (6)

^a^Only one T1D service is available for people aged up to 18 years in the following jurisdictions: Western Australia, the Northern Territory and the Australian Capital Territory.

Most respondents provided care in multiple settings: 15 (46%) in two settings and 14 (43%) provided care in three to five settings. Providers worked mostly in paediatric outpatient clinics (*n* = 28, 88%) and/or in inpatient clinics in general hospitals (*n* = 21, 66%). The total number of children and young people with T1D seen annually in metropolitan services ranged from 60 in the Northern Territory to 1200 in Western Australia and from 4 to 150 patients per year in regional services. Collectively, the services managed 6669 patients per year.

Most services (*n* = 26, 81%) saw patients three or four times per year. Two providers reported adjusting the frequency of visits according to need; for example, patients who had trouble complying with their treatment plan or had ongoing challenges with glycaemic control were seen more frequently. The duration of a consultation varied among providers. Most reported 30 min or less when patients were seen by a doctor (*n* = 23, 72%), a dietician (*n* = 19; 53%), a diabetes educator (*n* = 19; 53%) or a diabetes nurse (*n* = 16, 72%). Two regional services reported 90‐min appointments (Table 2).

**Table 2:** Service practices by location.

Service practices	Major city, *n* (%)					Regional, *n* (%)				
Duration of consultation (time)	≤30 min	60 min	90 min	HP not available		≤30 min	60 min	90 min	HP not available	
Doctor	13 (81)	3 (19)	— (0)	— (0)		10 (63)	6 (38)	— (0)	— (0)	
Diabetes nurse	9 (56)	1 (6)	— (0)	6 (38)		7 (44)	2 (13)	1 (6)	6 (38)	
Dietician	10 (63)	6 (38)	— (0)	— (0)		9 (56)	7 (44)	— (0)	— (0)	
Diabetes educator	10 (63)	6 (38)	— (0)	— (0)		7 (44)	8 (55)	1 (6)	— (0)	

Co‐management with other HP (%)^a^	0%–10%	10%–20%	20%–30%	30%–40%	90%–100%	0%–10%	10%–20%	20%–30%	30%–40%	90%–100%
	12 (75)	2 (13)	— (0)	1 (6)	1 (6)	12 (75)	— (0)	2 (13)	— (0)	2 (13)

Links with primary health network	Yes		No	Don’t know		Yes		No	Don’t know	
	3 (20)		9 (60)	3 (20)		2 (13)		12 (75)	2 (13)	

Out‐of‐hours service available	Yes		No			Yes		No		
	11 (69)		5 (31)			8 (50)		8 (50)		

*Note:* Totals may not equal 100% due to rounding.

^a^Co‐management scale was 0%–100% in 10% increments. Only increments with responses are shown in the table.

##### 3.1.2.1. Co‐Management and Links to Primary Health Networks and General Practitioners

Co‐management of patients with other services was uncommon, with 24 clinics (75%) reporting co‐managing 10% or less of their patients. However, three clinics (one each in remote Queensland, a large city in New South Wales (NSW) and regional Victoria) co‐managed 90%–100% of their patients (Table 2).

Five services (16%) were linked with their local Primary Health Network, mostly by providing educational opportunities for general practitioners (GPs) (Table 2). Two T1D services supported a GP HealthPathway [21] for T1D, and one service in regional Victoria held regular interdisciplinary meetings that included GPs. The main means of communication with GPs was via a letter generated from the outpatient clinic or a discharge summary after hospital admissions (*n* = 29, 91%). One respondent also communicated with GPs via regular meetings/telephone calls.

##### 3.1.2.2. Out‐of‐Hours Services

Nineteen (59%) services provided out‐of‐hours services, and 12 of the 19 (63%) were located in large cities (Table 2). Thirteen of the 19 respondents (68%) provided additional information: these out‐of‐hours services consisted mainly of telephone access to a paediatrician, an endocrinologist, a doctor‐in‐training or a diabetes nurse educator. Covered out‐of‐hours periods varied, with eight services providing 24/7 telephone access, whereas others provided access only during certain times (e.g., 8 am–8 pm or until 10 pm) or for certain groups of patients (e.g., accessible only to patients newly transitioned to pump therapy, or newly diagnosed patients).

##### 3.1.2.3. Psychosocial and Educational Support

Eight services (29%, major cities: *n* = 4, regional/rural: *n* = 4) reported having at least one dedicated psychologist on the T1D team (two in NSW, two in Queensland, two in Tasmania, one in South Australia and one in Western Australia). The wait times to see a psychologist ranged from 24 h to 2 months with 63% reporting wait times of less than 1 month. One clinic in regional Tasmania had funding for a psychologist but did not fill the position due to the lack of appropriately qualified candidates.

Sixteen services (57%, eight in a major city) provided patients with information about local T1D support groups accessible via social media (*n* = 7, 22%), in person (*n* = 1, 3%) or both (*n* = 8, 25%). Twelve services offered patients and families access to mobile health information technology (42%), including MyLife and Calorie King apps (*n* = 9) and text messaging services (*n* = 4).

Of the 26 services responding, 22 (85%) offered education programmes for patients or families as part of their ongoing T1D care (Table 3). The most common programmes covered transition to adult services, carbohydrate counting, sick day management and use of diabetes technology (Table 3).

**Table 3:** Types of education programmes offered as part of ongoing care (total respondents *n* = 26).

Type of programme provided	Number of clinics (*n*)	%	Metropolitan/regional (*n*)
Diabetes and transition to adult services	17	65.4	11/6
Nutrition and carbohydrate counting	17	65.4	10/7
Diabetes and sick day management	15	57.7	8/6
Diabetes technologies	15	57.7	9/6
Insulin adjustment	14	53.8	9/5
Drugs and alcohol	11	42.3	8/3
Diabetes and driving	11	42.3	8/3
Diabetes and exercise	10	38.5	6/4
Diabetes and smoking	9	34.6	7/2
Pre‐school to school transition preparation	9	34.6	4/5
Diabetes and pregnancy	8	30.8	4/4
Preparation for independent travel after leaving high school	6	23.1	4/2
Programmes offered by other organisation, e.g., Diabetes Australia	4	15.4	1/3

##### 3.1.2.4. Telehealth and the COVID‐19 Pandemic

Twenty‐one clinics (66%) provided telehealth consultations prior to the COVID‐19 pandemic, compared with 32 (100%) post‐pandemic (McNemar, *χ* ^2^(1) = 11.000, *p* < 0.001, Cramér’s *V* = 0.58). The most common reason for currently using telehealth was COVID‐19 or another infectious disease (*n* = 27, 84%), family not being able to access transport to attend in person (*n* = 22, 69%) or long distance to the clinic (*n* = 22, 69%). Six providers (19%) reported using telehealth consultations because of patient and/or carer mobility issues or having an immunocompromised patient or carer. One provider offered telehealth as an out‐of‐hours service only.

Twenty‐six (81%) respondents provided examples of perceived advantages and disadvantages of using telehealth (Table 4).

**Table 4:** Perceived advantages and disadvantages of telehealth vs. face‐to‐face consultations.

Perceived advantages	Perceived disadvantages
Facilitates greater access, engagement, and appointment attendance (virtual)	Limited physical examination and team‐based care
• Eases access for patients who live far from the clinic • Brings care closer to home • Increases engagement and frequency of contact when needed • Provides greater flexibility and more frequent access to care when needed • Enables people to attend despite adverse weather or transport problems • Allows engagement even when the patient feels unwell	• Inability to check HbA1c levels, height, weight, or blood pressure • Limited point‐of‐care testing • Attendance of only one health professional rather than the whole team • Difficulties managing psychosocial issues, complexities of problems and referrals
Advantages for families	Harder to develop rapport:
• Reduces time off work • Reduces time off school for child with T1D and siblings • Provides greater access to education and upskilling in new technologies (pumps, CGMs) • Enables capacity‐building and confidence for managing T1D, including new technologies, while being flexible	• Preference for telephone over video • Difficult, complex circumstances for families creating privacy/confidentiality challenges • Time poverty of families who take telehealth consultations “on the run” and cannot engage properly • Greater difficulty of picking up nonverbal cues via telehealth • Especial difficulty engaging with the child or adolescent with T1D
Financial	Financial
• Reduces lost income and out‐of‐pocket expenses for families (e.g., travel costs, parking costs)	• Families living with socioeconomic disadvantage prefer telehealth, but telehealth is helpful only in certain circumstances
Helpful but only in certain circumstances	Technical issues
• Easier to engage • High‐functioning families • Families without psychosocial complexity • Families who have good glycaemic control and need minor adjustments • Good for in between face‐to‐face consultations	• Downloaded clinical data often not available at time of consultation • Family difficulties in uploading pump and CGM data without support • Limited internet access especially for rural or remote families

*Note:* Categories and subcategories were generated through content analysis of the free text responses.

#### 3.1.3. Types of Health Professionals Available in T1D Services Across Australia

Thirty‐one service leaders responded to the workforce section of the survey (major cities *n* = 15; regional/remote *n* = 16). Most services (*n* = 20, 64%) had a mix of full‐ and part‐time staff (Table 5). Four regional services reported no full‐time staff (25%) (Table 5). None of the services reported having both full‐time and part‐time staff in the same profession.

**Table 5:** Health professionals by employment status (full‐time or part‐time) across the T1D services.

Health professional staff	All services (*n* = 31^a^)			Major city (*n* = 15)			Regional (*n* = 16)		
	Total, *n* (%)	Full time, *n*	Part time, *n*	Total, *n* (%)	Full time, *n*	Part time, *n*	Total, *n* (%)	Full time, *n*	Part time, *n*
Doctors									
Endocrinologist	7 (23)	3	4	3 (20)	1	2	4 (25)	2	2
Paediatric endocrinologist	19 (61)	7	12	15 (100)	7	8	4 (25)	0	4
General paediatrician	21 (68)	4	17	6 (40)	0	6	15 (94)	4	11
Fellow/Registrar	10 (32)	5	5	8 (53)	5	3	2 (13)	0^b^	2
Nurses									
Hospital nurse practitioner	5 (16)	5	0	4 (27)	4	0	1 (6)	1	0
Clinical nurse consultant	15 (48)	12	3	11 (73)	9	2	4 (25)	3	1
Clinical nurse specialist	22 (71)	15	7	13 (87)	8	5	9 (56)	7	2
Community nurse practitioner	1 (3)	1	0	1 (7)	1	0	0 (0)	0^b^	0
Allied health professionals									
Dietitian	29 (94)	10	19	14 (93)	6	8	15 (94)	4	11
Social worker	17 (55)	4	13	10 (67)	4	6	7 (44)	0^b^	7
Psychologist	9 (29)	1	8	5 (33)	1	4	4 (25)	0^b^	4
Podiatrist	4 (13)	1	3	1 (19)	0	1	3 (19)	1	2
Physiotherapist	1 (3)	1	0	0 (0)	0	0	1 (6)	1	0

*Note: n* = Number of services reporting.

^a^One service did not complete the question about full‐ or part‐time staff.

^b^Four regional services reported no full‐time staff.

The team composition varied greatly, with eight services (26%, six in major cities) reporting staff in seven out of 13 different professions (Table 5). The most common types of professionals working in the T1D teams were dieticians (*n* = 29, 94%), clinical nurse specialists/diabetes educators (*n* = 22, 71%), general paediatricians (*n* = 21, 68%) and paediatric endocrinologists (*n* = 19, 61%) (Table 5). Overall, nurses tended to be employed full‐time, whereas doctors and allied health professionals tended to be part‐time (Table 5).

All services located in major cities reported having a paediatric endocrinologist (*n* = 15, 100%), compared with 25% of the regional or remote services (*n* = 4; *χ* ^2^ = 18.355; *p* < 0.001, Cramér’s *V* = 0.77). Services in major cities were more likely to include clinical nurse consultants (major city: *n* = 11, 73%; regional/rural: *n* = 4, 25%; *χ* ^2^ = 7.2419; *p* < 0.05, Cramér’s *V* = 0.48) and clinical nurse specialists (major city: *n* = 13, 87%; regional/rural: *n* = 9, 56%); however, this difference was not significant (Table 5). Four regional/rural clinics reported lacking access to nursing staff; however, they had full‐ or part‐time allied health professionals, primarily dietitians.

Twenty‐one services (68%) reported that patients had access to MDTs during a single consultation (major city *n* = 9, regional/remote *n* = 12). All clinics reported trying to ensure that patients saw the same clinical team every visit (*n* = 12, 39%) or when possible (*n* = 19, 61%).

### 3.2. Interview Results

#### 3.2.1. Provider Characteristics

Twenty‐two clinicians (69%) indicated a willingness to participate in a follow‐up interview. However, only seven clinicians (four physicians and three nurses from four states) ultimately participated in the interviews. Three clinicians from Tasmania opted to be interviewed together and were not recorded. Five clinicians provided services in a large city (Tasmania, Western Australia and NSW) (Supporting Information 2: Supplemental Documentation Table S1) and a detailed thematic analysis including illustrative quotes is provided in Supporting Information 3: Supplemental Documentation Table S2.

#### 3.2.2. The Value of Teams and Data Infrastructure

Two major themes emerged on what was functioning well in the interviewees’ service: *teams and staff* and *data infrastructure and tools*.

Five clinicians discussed the value of a collaborative, skilled MDT with diabetes‐specific knowledge. The MDT structure supported family‐centred education and support to build patients’ capacity and confidence for self‐care. Low staff turnover meant consistency of care, strong team cohesion, supporting therapeutic relationships with patients and families:

*‘Low turnover of staff maintains consistency* [*for patients*]. *… see same doctor and services’* (*Provider 7*).

Clinicians valued staff capacity and established service agreements that facilitated equitable, consistent care delivery, including through outreach clinics in rural regions (Supporting Information 3: Supplemental Documentation Table S2).

*Data infrastructure and tools*, including tools such as SMS and emails with patients outside of scheduled consultations and remote monitoring of CGM data enabled timely responses to medical concerns:

*‘Communication with families*, *texts*, *emails*, *they look at patient’s CGM; patients are open about the data’* (*Provider 3*).

One clinician reported that clinic performance data were of significant value in guiding service improvements:

‘[*We have*] *reasonable quality data on outcomes* (*and infrastructure that supports this*). *We can collect baseline data on service performance to track over time and measure influence on outcomes’* (*Provider 4*).

#### 3.2.3. Challenges of Service Provision and Suggested Improvements

The main challenges clustered around three themes: 1. *Coordinated care across health providers*, 2. *Composition of teams and staff availability* and 3. *Inequitable access to care for patients and families*.

T1D specialists identified challenges engaging with GPs, with two clinicians cited increased workload in the specialist T1D clinic when patients and their families expected care for non‐diabetes‐related concerns that could be covered by GPs (Supporting Information 3: Supplemental Documentation Table S2). Shortages of full‐time staff acrosss the specialist T1D services resulted in increased patient waiting times, reduced the ability to conduct outreach clinics and limited capacity to provide flexible patient‐centred care. Limited access to psychologists, counsellors and social workers to support patients’ mental health and wellbeing was often discussed, and this aligned with the quantitative data reported in Table 3, above. Four clinicians identified the lack of psychological support for patients as a care quality and safety issue, as patients and families struggled to maintain glycaemic targets because their psychosocial difficulties remained unaddressed.

Shortages of clinical staff increased patient waiting times, reduced the ability to conduct outreach, and limited capacity to provide flexibility in care (*n* = 4). Such shortages also negatively impacted staff’s ability to keep up with educational training and professional development. Two providers expressed concerns about staff funding and the impact of part‐time staff on shared workloads (*n* = 2).

Three clinicians highlighted that international guidelines did not always consider contextual and resource factors in rural clinics needed to implement the guidelines:

*‘Compared with ISPAD recommendations*, *need to be careful in setting the benchmark to clinics in the city. They* [rural clinics] *are also under-resourced so it would not be fair to look at them and decide what the regional clinic needs. For example*, *they might not have a psychologist on board but that does not mean that regional clinic does not need one.’* (*Provider 5*).

This reflects the quantitative data on limited access to paediatric endocrinologists, psychologists and social workers in regional clinics.

When asked about the one thing that needed to be changed to improve the care of T1D patients in their clinic, two themes emerged: *funding to increase staffing levels and health professional availability* and *funding for other support*.

Aligning with the quantitative data, six providers highlighted the need for adequate funding for diabetes‐trained psychologists who have capacity and time to support newly diagnosed patients and families. For example, Provider 6 said,

*‘Psychosocial support definitely. We have the medical staffing resources to look after glycaemic control, the science, and logistics. Don’t have the ability to support [patients] when in crisis, help them with motivation and burnout*.’

Additional funding to create an after‐hours medical team to support families when needed was also identified as a priority (Supporting Information 3: Supplemental Documentation Table S2).

Service leaders called for increased government funding to enable equitable access to T1D technology for all children and youth with T1D. They also highlighted the need for navigators/coordinators who can assist patients and families to access existing government‐funded assistance programmes such as Centrelink [22] and the National Diabetes Services Scheme [23] to improve patient outcomes and reduce the burden on specialist T1D health services.

### 3.3. Integration and Synthesis of Survey and Interview Findings

Qualitative interviews supplemented the survey findings with more nuanced insights into how the clinics functioned and areas for improvement. Both sources of data identified the importance of the MDT approach. The qualitative data highlighted the value that T1D service leaders placed on having access to highly skilled, stable and cohesive MDTs. However, reliance on part‐time staff, evident from the survey and strengthened by the interview themes, indicated that many clinics struggle to meet demand for care because of staff shortages and small full‐time equivalent fractions allocated to the clinics.

The need to deliver integrated care that included psychological and primary care support was a consistent finding from both the survey and interviews. Interviews provided more details on the challenges posed by the lack of embedded psychologists in the MDT, including long waiting times for appointments and limited opportunities for psychologists to build T1D‐specific skills. Limited communication channels and lack of integration of GPs with the T1D MDTs were seen as lost opportunities for greater care integration. In addition, families’ expectations of receiving non‐T1D‐related care from the specialist MDT rather than from a GP created additional workload for the MDT. The potential value of primary care HealthPathways [21] to improve continuity of care was evident; however, T1D service leaders expressed doubt about the level of implementation of HealthPathways in primary care clinical practice [24, 25].

## 4. Discussion

Adopting a mixed‐methods approach, this study addressed a gap in knowledge about the current models of care for Australian children and youth living with T1D. The survey captured data from clinics that provided care to over half of the estimated 13,200 people under the age of 19 living with T1D in Australia. Consistent with the ISPAD guidelines of having specialists with training in T1D and paediatrics as part of the multidisciplinary care team [26], our results show that all of the surveyed services reported having an MDT or having access to an MDT. However, many MDTs relied on a mix of part‐time medical, nursing and allied health staff; 32% of surveyed services had just one full‐time staff member. Services in major cities were more likely to have embedded full‐time senior nursing positions (clinical nurse specialists and clinical nurse consultants), which provided needed core capacity for patient management, especially when most other staff, including doctors and allied health professionals, were employed part‐time. Significantly, fewer regional/rural services had a paediatric endocrinologist on staff, and many clinics in large cities provided outreach to boost access to paediatric endocrinology expertise in regional areas. Most of the service leaders surveyed worked in more than one clinic. The combination of insufficient staff numbers and high proportions of part‐time staff created challenges in providing timely, person‐centred care, especially when some patients and families needed additional support. Despite staffing challenges, 68% of clinics reported that patients were able to engage with multiple team members during consultations, taking a multidisciplinary care approach, in line with the ISPAD guidelines [26]. However, this means that in one‐third of the clinics surveyed, this was not possible.

As diabetes‐related distress and other psychosocial challenges are known to affect the ability of patients and families to maintain glycaemic control [27–29], the ISPAD guidelines specifically recommend embedding a diabetes‐specialised psychologist in the MDT [26]. However, service providers highlighted the lack of dedicated psychologists in Australian MDTs as a significant gap in service capacity. The lack of psychological support increases risks of developing comorbidities known to occur with T1D, including diabetes‐related distress, anxiety and eating disorders [9]. In the absence of a dedicated psychologist, clinics often referred patients to general psychological services in children’s hospitals (which have long wait times and limited access to T1D‐trained psychologists) or to private psychologists (which increased time and cost burdens). Patients were also referred to GPs for the creation of publicly funded mental health plans that cover the cost of a limited number of sessions with a psychologist [30]. However, these referral pathways tend to be disjointed and limit care integration with the MDT. Integrating psychologists within T1D teams brings value by improving glycaemic control and supports better physical and mental health outcomes among children and youth with T1D [31]. Boosting T1D training for psychologists and building psychological workforce capacity are also needed, which is aptly illustrated by one service where a position for a psychologist was funded, but recruiting an appropriately skilled candidate was problematic [11, 31].

Involving GPs more closely with specialist T1D teams has potential benefits for managing children and youth with T1D, enabling GPs to provide care and support in between visits to specialist T1D clinics [26]. In our study, providers reported limited connections with GPs, communicating mostly through the exchange of letters or hospital discharge summaries. This reflects the unintegrated structure of the health system in Australia, where primary care is administered and funded separately from hospital‐based services [32]. However, providers indicated that greater connection with GPs would bring value to their patient‐centred MDTs whilst building capacity among GPs to care for children and youth with T1D. Evidence from an Australian study showed that GPs find recognising and managing T1D in children and youth challenging [30]. The development of HealthPathways (online portals for GPs that provide information on the assessment and management of specific conditions) could facilitate referral and communication between GPs and T1D specialist teams [21, 33]. However, previous research shows limited use of HealthPathways in clinical practice [24, 34]. The extent to which GPs access and apply T1D‐specific HealthPathways in their clinical practice is currently unknown. Adapting approaches used successfully to improve care quality in primary care for other conditions, including T2D, where specialist teams are linked closely with GPs, could be adapted for T1D care [35, 36]. Furthermore, establishing multidisciplinary communities of practice using well‐established platforms such as Project ECHO could support practical case‐based learning to enhance interdisciplinary collaboration and care integration across the primary and specialist care sectors [37, 38].

The ISPAD guidelines also recommend the use of telehealth as a means of connecting patients with their MDT [26]. The use of telehealth by T1D clinics has increased significantly since the start of the COVID‐19 pandemic, and providers have identified some advantages, for example, convenience and ease of access for providers and families. However, some also expressed concerns related to technical issues, limits to physical examination or pathology collection, privacy issues and difficulties developing rapport and relationships with patients and families [39, 40].

This research provides a benchmark of the current service practices and team composition. It identifies from the service leaders’ perspective aspects of the model of care that are fit for purpose and areas for improvement. This type of information is essential for prioritising improvements for Australian services and to improve equitable access across currently under‐served populations [41]. The multidisciplinary models of care recommended by ISPAD, which include access to psychological support and 24 h access to care, have been implemented to varying degrees across Australia. Clinical leaders clearly recognise the problem of limited access to psychological support and 24 h access to care; however, service planners and funders have not acted upon this, with funding for services being a major problem reported by most services regardless of location [41]. The recommendation of providing care coordinators to help families navigate the complexities of health and social care systems needed to effectively manage T1D in Australia should be considered. Care coordination is key component of patient‐centred care and could be incorporated as part of a multidisciplinary care T1D framework [42, 43].

Furthermore, service leaders across Australia identified specific concerns about equity of access to essential T1D technology, including CGM and insulin pumps. Although the Australian Government expanded subsidies for CGM to include all Australians with T1D from July 2022 [44], insulin pumps are still not government‐funded for all children and youth. The lack of subsidised insulin pumps limits access for families who do not have the right level of private health insurance or who are otherwise socioeconomically disadvantaged. This identified gap in equitable access prompted a government inquiry and subsequent report recommending universal access to government‐funded insulin pumps for children and youth living with T1D [45]. As the management of T1D changes, including new screening programmes for the early identification of pre‐diabetes and disease‐modifying trials, current T1D models of care will need to be continually adapted and updated [46, 47]. Research will be needed to understand how these programmes would affect an already stretched workforce and to understand what new roles or care pathways may be needed.

Although international guidelines are helpful in outlining optimal models of care, our findings highlight the importance of designing models of care that are flexible, adaptable to local conditions and fit‐for‐purpose for diverse locations and populations. In particular, policy and funding changes are needed to enable access to outreach and telehealth models of care that involve multiple specialist clinicians and local primary care providers, especially for children and youth living in regional, rural and remote areas of Australia (Box 1) [48, 49].

**Box 1: Policy Implications.**
• Provide additional funding for embedded T1D‐trained psychologists in MDTs.
• Increase training courses for psychologists, GPs and other health professionals (e.g., care coordinators) to enable greater support of patients with T1D and their families in between visits to the specialist MDT.
• Increase funding for full‐time staff in regional services to boost MDTs and build capacity for more equitable access to specialist care across geographical regions.
• Increase funding for out‐of‐hours care to ensure support for patients and families when needed.
• Create and fund care coordinator positions to facilitate communication between families, MDTs and other health professionals (e.g., GPs, allied health professionals).
• Fund insulin pumps for all children and young people living with T1D to improve equity of access to technology.
• Greater funding for telehealth consults with multiple health professionals.

### 4.1. Study Strengths and Limitations

Strengths of this study include being the first Australia‐wide survey of T1D services, capturing survey data from service leaders from all states and territories, spread across major cities and regional/rural areas and including responses from major tertiary centres in most states as well as capturing data from services that care for over half of people 19 years and younger living with T1D in Australia. The mixed‐methods approach enhanced the depth of interpretation with further nuanced understanding of the current contexts of T1D services and service leaders’ experience of service gaps and identification of priorities for improvement. The information presented here provides an important description of the models of care as implemented in Australia in 2022. This survey could be repeated to determine progress towards greater adoption of MDTs in Australia and internationally as policies, T1D technology and demands for care change over time [26]. Keeping abreast of such changes is crucial for model of care optimisation, including workforce planning, recruitment and upskilling. As the evidence for early screening, monitoring and interventions increase for early‐stage diabetes before symptoms are apparent models of care and referral pathways may also need to be enhanced to deliver appropriate care during this early disease phase [50].

Although all major T1D services across all states were approached to participate, only two service leaders responded from the state of Victoria (both were from regional/rural services), creating a gap in the collected data about Victorian T1D services in major cities. In contrast, services from Queensland were over‐represented, potentially creating a bias. Queensland has an established state‐wide T1D clinical network, which streamlined survey recruitment. The organisation of services in Queensland is also less centralised with greater outreach and connection between tertiary specialist centres and services in regional/rural areas. However, the overall model of service is similar to other states and territories. Moreover, the data from other states from both regional/rural and major city services aligned with the data gathered from Queensland, suggesting that the results are applicable across locations. Recruitment through professional networks (e.g., ANZSPED/APEG and JDRF) may have attracted more engaged or better‐resourced services. Smaller or underfunded clinics may be under‐represented, which could skew results toward more favourable workforce and technology access.

The lack of available clinical outcome data to accompany the analysis of health service models of care in this study is a limitation. A national database of clinical outcomes that is accessible to researchers is very much needed. This would also support health economic analyses to determine the value of different models of care operating in different contexts.

Although the survey captured most specialist paediatric clinics operating in an outpatient context in major children’s hospitals, many paediatric clinics established in general hospitals are unlikely to have been captured. This gap should be addressed in future studies.

Recognising that service providers have limited time for participating in research surveys, the survey was restricted in length and detail and co‐designed with a group of clinicians to increase relevance. Despite this, 10 respondents (31%) only partially completed the full survey. Overall, the questions included in this article averaged a 93% (*n* = 30) response rate (Range: 81% *n* = 26% to 100%, *n* = 32, Mode: 97%, *n* = 31). The themes generated from the limited number of follow‐up interviews may not completely reflect the experiences and opinions of other service leaders; however, the comments provided aligned well with the responses to the survey questions, while providing further nuanced information. Engaging clinicians in interviews is challenging due to constraints on their time and potential loss of income for time‐intensive research. Better compensations for clinicians for participation should be considered in the future.

## 5. Conclusion

Using a mixed‐methods approach, this study comprehensively describes the T1D services available in Australia for children and youth. The current models of care are responsive to the ISPAD recommendations for care provision via an MDT approach. However, significant areas for improvement were identified, including increasing and expanding service capacity, particularly through the inclusion of diabetes‐trained psychologists and promoting greater connections with primary health services. Service leaders also identified inequitable access to T1D services in regional/rural areas and inequitable access to T1D technology—priorities that still need to be urgently addressed. The results reveal the need for flexibility in implementing clinical models of care across the widely varied Australian geography with dispersed populations and limited funding and resources. Including telehealth consultations and outreach clinics that bring specialists from major cities into closer clinical collaboration with clinicians working in regional/rural areas is an area of strength that could be further developed through national clinical networks and communities of practice. The results provide a crucial baseline for understanding how changes in policies and technologies may impact the health workforce and healthcare delivery in the future.

## Author Contributions

**Yvonne Zurynski**: conceptualisation (equal), funding acquisition (equal), methodology (equal), formal analysis (equal) writing – review and editing (equal). **Carolynn L. Smith:** data curation (lead), formal analysis (equal), visualisation, writing – original draft (lead), writing – review and editing (equal). **Ann Carrigan:** methodology (equal), investigation (lead), writing – original draft (supporting), writing – review and editing (equal). **Nehal Singh:** investigation (supporting), data curation (supporting), writing – review and editing (supporting). **Timothy W. Jones:** conceptualisation (equal), funding acquisition, methodology (equal), writing – review and editing (equal). **Leanne Cromb:** methodology, writing – review and editing (equal). **Helen J. Woodhead:** methodology, writing – review and editing (equal). **Anthony Pease:** methodology (equal), writing – review and editing (equal). **Tony Huynh:** funding acquisition (equal), methodology (equal), writing – review and editing (equal). **Ann M. Maguire:** funding acquisition (equal), writing – review and editing (equal). **Kristen A. Neville:** investigation (supporting), writing – review and editing (equal). **David E. Bloom:** funding acquisition (equal), writing – review and editing (equal). **Sophia Zoungas:** funding acquisition (equal), writing – review and editing (equal). **Jenny Couper:** funding acquisition (equal), methodology (equal), writing – review and editing (equal). **Jeffrey Braithwaite:** conceptualisation (equal), funding acquisition (equal), writing – review and editing (equal). **Elizabeth A. Davis:** conceptualisation (equal), funding acquisition (equal), methodology (equal), writing – review and editing (equal).

## Funding

This project was supported by the Breakthrough T1D (Grant #5_SRA‐2021‐1088_M‐X). Open access publishing facilitated by Macquarie University, as part of the Wiley ‐ Macquarie University agreement via the Council of Australasian University Librarians.

## Disclosure

The funding source played no role in the design, collection, analysis, or interpretation of the data or in the decision to submit for publication.

## Ethics Statement

This study was performed in accordance with the Declaration of Helsinki and approved by the Faculty of Medicine, Health and Human Sciences Research Committee, Macquarie University (#520221154439676). Participants received an information sheet, and informed consent was obtained from all participants.

## Consent

Participants provided written consent in accordance with the HREC‐approved Patient Information and Consent Form, which detailed how the findings would be disseminated.

## Conflicts of Interest

The authors declare no conflicts of interest.

## Supporting Information

Additional supporting information can be found online in the Supporting Information section.

## Supporting information


**Supporting Information 1** Supporting Information File 1: Survey questions administered via REDCap [16].

**Supporting Information 2** Supplemental Documentation Table S1: Provider role, service location and setting (*n* = 7).

**Supporting Information 3** Table S2: Thematic analysis summary.

## Data Availability

The data that support the findings of this study are available upon request from the corresponding author. The data are not publicly available due to privacy or ethical restrictions.
