# Supplementary material for: Health Service Leaders’ Perspectives on Type 1 Diabetes Models of Care for Children and Young Adults in Australia: A Mixed‐Methods Study
Source: J Diabetes Res. 2026 Apr 29;2026:7441677. doi: 10.1155/jdr/7441677 (PMC13128981; doi:10.1155/jdr/7441677)
Supplement: Supplementary file 1 — Supporting Information 1 Supporting Information File 1: Survey questions administered via REDCap [16]. [file JDR-2026-7441677-s001.pdf]

Supplemental File 1

Healthcare Provider Survey

Participant Information and Consent FormChief Investigator: Associate Professor Yvonne Zurynski
Email: Yvonne.Zurynski@mq.edu.au
Project Title: Investigating T1D Models of Care, Implementation Science and Research Translation
Funding Body: Juvenile Diabetes Research Fund
HREC Reference: 11544
Chief Investigator: Associate Professor Yvonne Zurynski
Co Investigators:
Dr Ann Carrigan
Professor Elizabeth Davis
Professor Tim Jones
Professor Sophia Zoungas
Associate Professor Tony Huynh
Professor Jenny Crouper
Professor Jeffrey Braithwaite
Ms Nehal Singh
Ms Leanne Cromb
Ms Sophie Lumby

We ask that the most senior person from the service complete the survey, such as the clinical lead. If you work in multiple services, please fill out for the largest and kindly forward the survey on. You will have the options to save your responses and return at a later stage if required to.

You are invited to participate in this study exploring existing models of care for individuals living with Type 1 Diabetes in Australia. This research will scope and map how care is provided, both for children and young adults living with Type 1 diabetes, investigating what current services are available and utilised, barriers and enablers, and resourcing of services in Australia. The research will guide future improvements to these healthcare services.

This survey focuses predominantly on Type 1 diabetes services. There are questions specific to Type 2 diabetes at the end of the survey.

The study is being conducted by Associate Professor Yvonne Zurynski and Dr Ann Carrigan from the Australian Institute of Health Innovation at Macquarie University. If you have any questions please contact Dr Ann Carrigan (phone: 0414692204 email: ann.carrigan@mq.edu.au). The research project is being conducted by Macquarie University in partnership with the Rio Tinto Children's Diabetes Centre; a JDRF Global Centre of Excellence and is funded by JDRF Australia.

As a healthcare provider you are being invited to participate in this survey, which should take no longer than 45 minutes to complete. Participation in this study is entirely voluntary: you are not obliged to participate and if you decide to participate, you are free to withdraw at any time. Information about your service will be confidential and de-identified and no information which is published or shared will identify you or your service in any way. Services will be identified by location (metro/rural) and whether hospital based, inpatients or outpatients or private clinic/rooms based. Your responses will only be used for the purpose of this research project and they will only be disclosed with your permission, except as required by law.

At the end of the survey, you will be asked if you would like to be part of a team reviewing the findings and developing a manuscript.

At the end of the survey you may elect to enter a draw to win a $200 Booktopia voucher as a thank you for your participation.

A summary of the report after analysis and published papers can be provided after the research has concluded, if requested.
If you agree to participate, please select "I accept" below to continue to the survey.

I have read and understood the above information, and any questions I have asked have been answered to my satisfaction. I agree to participate in this research, knowing that I can withdraw from participation in the research at any time without consequence.	◯ I accept<br>◯ I do not accept

1. How would you describe your clinical role?

- ◯ General paediatrician
- ◯ Paediatrician with special interest in T1 diabetes
- ◯ Paediatrician with special interest in T1 diabetes and endocrinology
- ◯ Paediatric diabetologist or endocrinologist
- ◯ Adult endocrinologist consultant
- ◯ Adult endocrinologist fellow
- ◯ Adult endocrinologist registrar
- ◯ Diabetes nurse practitioner
- ◯ Diabetes nurse
- ◯ Diabetes educator
- ◯ Community nurse
- ◯ Dietician
- ◯ Psychologist
- ◯ Exercise physiologist
- ◯ GP (if linked to a paediatric diabetes team)
- ◯ Other (please specify below)

1a. If you selected "other" above, please enter your profession here.

____

2. What is your age?

- ◯ < 31
- ◯ 31-35
- ◯ 36-40
- ◯ 41-45
- ◯ 46-50
- ◯ 51-55
- ◯ 56-60
- ◯ >61

3. What is your sex?

- ◯ Male
- ◯ Female
- ◯ Other/ Prefer not to say

4. What is the location or postcode of your clinic?

____

The following questions are meant to determine details of routine Type 1 diabetes care services offered by your service.

4a. In what setting(s) does your service see children or young adults with Type 1 diabetes? (select all that apply)	☐ Young adult diabetes service<br>☐ Paediatric diabetes outpatient clinic<br>☐ Paediatric inpatients in a paediatric hospital<br>☐ Paediatric inpatients in a general hospital<br>☐ General diabetes outpatient clinic in an adult hospital<br>☐ Community outreach outpatient clinic<br>☐ Large regional clinic (and inpatient setting)<br>☐ Rural clinic<br>☐ Private clinic<br>☐ In patient's home<br>☐ Other, please specify below
4b. If the location at which you treat children and young people with T1D was not listed above, please specify here.	__________________________________
5. How often do you usually see your T1D patients per year in a formal clinic?	__________________________________

The following questions ask about your clinics' patient numbers and whether you share care with other services. Estimates are fine.

6. Please estimate how many children and young people with T1D are seen each year in your service?

____

7. What proportion of patients seen by your service are co-managed with other services? For example, GPs. (Please enter a percentage)

- ◯ 0-10%
- ◯ 10-20%
- ◯ 20-30%
- ◯ 30-40%
- ◯ 40-50%
- ◯ 50-60%
- ◯ 60-70%
- ◯ 70-80%
- ◯ 80-90%
- ◯ 90-100%

8. When patients come to the service, if they see the doctor, on average how long do they spend with them?

- ◯ Thirty minutes or less
- ◯ One hour
- ◯ Ninety minutes
- ◯ Two hours
- ◯ More than two hours
- ◯ No Doctor at the service

9. When patients come to the service, if they see the diabetes nurse, on average how much time do they spend with them?

- ◯ Thirty minutes or less
- ◯ One hour
- ◯ Ninety minutes
- ◯ Two hours
- ◯ More than two hours
- ◯ No diabetes nurse at the service

10. When patients come to the service, if they see the dietician, on average how much time do they spend with them?

- ◯ Thirty minutes or less
- ◯ One hour
- ◯ Ninety minutes
- ◯ Two hours
- ◯ More than two hours
- ◯ No dietician at the service

11. When patients come to the service, if they see the diabetes educator, on average how much time do they spend with them?

- ◯ Thirty minutes or less
- ◯ One hour
- ◯ Ninety minutes
- ◯ Two hours
- ◯ More than two hours
- ◯ No diabetes educator at the service

12. Are interpreter services available on-site in your clinic?

- ◯ Yes
- ◯ No
- ◯ Don't know

12a. Why not?

____

13. Does the service offer children and young people with Type 1 diabetes and their families out-of-hours access (e.g., an advice line staffed by a member of the diabetes care team)?

- ◯ Yes
- ◯ No
- ◯ Don't know

13a. Please briefly describe the out-of-hours access that the service provides and who operates this service.	__________________________________
13b. Why not?	__________________________________
14. Do patients with T1D have access, if needed, to all team members at a clinic visit?	◯ Yes<br>◯ No<br>◯ Don't know
14a. Why not?	__________________________________
15. Does the service deliver care for T1D in other locations in addition to the main clinic, e.g. outreach clinics in rural regions or interstate?	◯ Yes<br>◯ No<br>◯ Don't know
15a. What is/are the name of the town/s where the clinic is held?	__________________________________
15b. How regularly does your service conduct these in these locations?	__________________________________
15c. Does the outreach clinic have a specific focus on complex cases of T1D?	◯ Yes<br>◯ No<br>◯ Don't know
15d. What is the total number of T1D patients seen per annum in the outreach service? (Estimates are fine)	__________________________________
15e. Do you have access to a specialist team when needed if you are a regional service?	◯ Yes<br>◯ No<br>◯ Don't know
16. How many newly diagnosed T1D patients does your service educate at diagnosis and then return to their local area for follow up, per annum?	__________________________________
17. In terms of population, how large is your catchment area? (e.g. state-wide service, Local Health District or Network, specific local region)	__________________________________

18. Who is routinely available in your diabetes service or via referral?

	Allocated to your service full time (clinical staff)	Allocated to your service part time or casual	Need to refer out	Not available / accessible
Endocrinologist	☐	☐	☐	☐
Paediatric Endocrinologist	☐	☐	☐	☐
Fellows/Registrars	☐	☐	☐	☐
Hospital Nurse Practitioners	☐	☐	☐	☐
Clinical Nurse Consultants	☐	☐	☐	☐
Clinical Nurse Specialists	☐	☐	☐	☐
Community Nurse Practitioners	☐	☐	☐	☐
Podiatrist	☐	☐	☐	☐
Orthodontist	☐	☐	☐	☐
Psychologist	☐	☐	☐	☐
Dietitian	☐	☐	☐	☐
Social Workers	☐	☐	☐	☐
Exercise physiologist	☐	☐	☐	☐
Physiotherapist	☐	☐	☐	☐
General paediatrician	☐	☐	☐	☐

19. Do you try to ensure that your T1D patients see the same clinical team at each visit?

○ Yes, always
○ Yes, when possible
○ No

20. Does your service link-in with the local Primary Health Network?

○ Yes
○ No
○ Don't know

20a. How is your service linked to the Primary Health Network?

☐ Provide educational opportunities for GPs
☐ Hold regular interdisciplinary meetings
☐ We have a GP Health Pathway for T1D
☐ Other (please specify below)

20b. Other (please specify)

____

21. How does your service communicate with patients GPs?

☐ Regular letter from OPD and a discharge summary for admissions
☐ Joint consult with GPs or regular/meetings/phone calls
☐ No regular contact
☐ Other (please specify)

21a. Other (please specify)

____

22. Do you offer T1D services specifically designed for the following populations? (Select all that apply)

☐ Rural and remote communities
☐ Aboriginal and Torres Strait Island peoples
☐ Pacifica
☐ Culturally and linguistically diverse
☐ Other (Please specify) below
☐ None

22a. Other (please specify)

____

23. Does the Type 1 diabetes team liaise regularly with school staff who supervise children and young people, to offer them diabetes education and information?

◯ Yes
◯ No
◯ Don't know

23a. If your service does not liaise regularly with school staff, why not?

____

24. Do you have access to the funded Diabetes Australia 'Diabetes In Schools' program?

◯ Yes
◯ No
◯ Don't know

24a. Why not?

____

25. Do you use the Diabetes Australia 'Diabetes In Schools' program?

◯ Yes
◯ No
◯ Don't know

26. Does your service offer patient reported experience measures (PREMs)?

◯ Yes
◯ No
◯ Don't know

26a. Which PREMS does your service measure?

____

27. Does your service offer patient reported outcome measures (PROMs)?

◯ Yes
◯ No
◯ Don't know

27a. Which PROMS does your service measure?

____

The next set of questions asks about the use of telehealth in your service pre and post COVID.

28. Pre-COVID, did your service use telehealth to conduct T1D consultations with patients / families? (select all that apply)

- ☐ Yes, mainly video consultations
- ☐ Yes, mainly telephone consultations
- ☐ Yes, we used video and telephone equally
- ☐ Yes, we sometimes used a hybrid of telehealth and face to face (e.g., the patient is face-to-face but their GP or another health professional is online or on the phone)
- ☐ No, we did not use telehealth

29. Currently, as part of your routine T1D care, on average across clinics, does your service use any of the following telehealth services to conduct consultations?

- ☐ Video consultations
- ☐ Telephone consultations
- ☐ Hybrid of video and telephone consultations
- ☐ No, Face-to-face only

29a. Currently, as part of routine T1D care, on average across clinics, what proportion of consultations in your service are video consultations? (Please enter a percentage)

- ◯ 0-10%
- ◯ 10-20%
- ◯ 20-30%
- ◯ 30-40%
- ◯ 40-50%
- ◯ 50-60%
- ◯ 60-70%
- ◯ 70-80%
- ◯ 80-90%
- ◯ 90-100%

29b. Currently, as part of routine T1D care, on average across clinics, what proportion of consultations in your service are telephone consultations? (please enter a percentage)

- ◯ 0-10%
- ◯ 10-20%
- ◯ 20-30%
- ◯ 30-40%
- ◯ 40-50%
- ◯ 50-60%
- ◯ 60-70%
- ◯ 70-80%
- ◯ 80-90%
- ◯ 90-100%

29c. Currently, as part of routine T1D care, on average across clinics, what proportion of consultations in your service are a hybrid of telphone and video as opposed to face-to-face? (please enter a percentage)

- ◯ 0-10%
- ◯ 10-20%
- ◯ 20-30%
- ◯ 30-40%
- ◯ 40-50%
- ◯ 50-60%
- ◯ 60-70%
- ◯ 70-80%
- ◯ 80-90%
- ◯ 90-100%

30. Please comment on the perceived advantages and disadvantages of using face- to-face and/or telehealth consultations

____

31. Under what circumstances would you typically use telehealth for T1D care? (Select all that apply)

- ☐ Accessibility issues for patient or family (mobility problems or other disability)
- ☐ COVID-19 or another infectious disease
- ☐ Immunocompromised patient or family
- ☐ Rural/remote family location
- ☐ The family cannot access transport
- ☐ Out of hours services only
- ☐ Government recommendation
- ☐ Never use telehealth
- ☐ Other (please specify below)

31a. Under what circumstances would you use telehealth? (Other)

____

Psychological support

32. Do you have a dedicated internal psychologist on site as part of your service?

◯ Yes
◯ No
◯ Don't know

32a. What is the average waiting time to see the psychologist in your service?

____

32b. What is the average waiting time for patients to see a mental health professional that you refer them to?

____

32c. If there is no dedicated internal psychologist as part of the service, to whom are mental health referrals made? (Select all that apply)

☐ Clinical psychologist
☐ Health psychologist
☐ Psychotherapist
☐ Psychiatrist
☐ Counsellor
☐ Mental health nurse
☐ Social worker
☐ Family therapist
☐ Other (please specify below)

32d. Other (please specify)

____

33. For what reasons do you refer patients to a psychologist?

	Yes	No	I would if the service was available
Problems with self-management	◯	◯	◯
Recurrent DKA	◯	◯	◯
Low BMI	◯	◯	◯
Eating disorders (bingeing, bulimia, anorexia)	◯	◯	◯
Disordered eating (e.g. skipping meals)	◯	◯	◯
Body dysmorphia	◯	◯	◯
Morbid obesity	◯	◯	◯
Depression	◯	◯	◯
Needle phobia	◯	◯	◯
Anxiety	◯	◯	◯
Drug and alcohol problems	◯	◯	◯
Psychotic illnesses	◯	◯	◯
Psychosexual health	◯	◯	◯
Domestic violence and/or sexual abuse	◯	◯	◯
Suicidal thoughts or self harm	◯	◯	◯
Family stress or trauma	◯	◯	◯
Communication problems with the family/team	◯	◯	◯
Poor diabetes adjustment	◯	◯	◯

34. What protocols does the service follow for children and young people with Type 1 diabetes in the following situations? (Select all that apply)

	Our own locally developed	State based protocols e.g. from the Department of Health	National	Aligned with APEG
Diabetic ketoacidosis	☐	☐	☐	☐
Hypoglycaemia	☐	☐	☐	☐
Surgery	☐	☐	☐	☐
Sick day management	☐	☐	☐	☐
Diabetes complications assessment management	☐	☐	☐	☐
Diabetes and exercise management for those on MDI and pumps	☐	☐	☐	☐
Insulin pump and CGM usage protocols	☐	☐	☐	☐
Diabetes in schools	☐	☐	☐	☐

35. If you use your own locally developed management protocols, are these? (Select all that apply)

☐ Local to your service
☐ Hospital-wide protocol
☐ Local health district/network
☐ State-wide protocol
☐ Does not apply

Diagnosis and initial management

36. Does your service use electronic medical records?

◯ Yes
◯ No
◯ Don't know

37. Does your service routinely enrol patients into a Type 1 diabetes registry? E.g., ADDN

◯ Yes
◯ No
◯ Don't know

37a. Which diabetes registry does your service enrol patients into? (select all that apply)

☐ Local
☐ ADDN
☐ SWEET
☐ Other (please specify)

37b. Other (please specify)

____

Management from diagnosis

38. Does the service develop Type 1 diabetes management plans or shared care plans?

◯ Yes
◯ No
◯ Don't know

38a. Who is given access to the management/ care plans? (Select all that apply)

☐ The team of health professionals at your service
☐ Health professionals to whom you refer patients
☐ The patient and/or family
☐ The GP
☐ The school
☐ Other (please specify below)

38b. Other (please specify)

____

39. If the plan is shared with the patient and/or family, how is this done? (Select all that apply)

☐ Through a web portal or app
☐ By email
☐ Through MyHealthRecord
☐ Printed and faxed/posted
☐ Care plans are not shared
☐ Other (please specify)

39a. Other (please specify)

____

40. Are children and young adults newly diagnosed with Type 1 diabetes offered a structured diabetes education program?

◯ Yes
◯ No
◯ Don't know

40a. Please provide details of structured education program

____

40b. Why aren't children and young adults offered a structured diabetes education program?

____

41. When do newly diagnosed T1D patients in your service usually commence on CGM?

◯ The first week
◯ Within the first 2 weeks
◯ 2 weeks to 1 month
◯ 1-2 months
◯ 2-3 months
◯ After 3 months
◯ It is up to the family

42. Are patients and/or families offered a choice of the CGM when first commenced?

◯ Yes
◯ No
◯ Don't know

42a. Why aren't families offered choice of the CGM when first commenced?

____

43. Where do most children and young adults receive care at the time of their T1D diagnosis? (Select all that apply)	☐ At home<br>☐ As an inpatient<br>☐ As an inpatient until they have insulin injection, BGL monitoring and hypoglycaemia management, then OPD education<br>☐ In ICU (DKA)<br>☐ In ICU (not DKA)<br>☐ Outpatient clinic<br>☐ Community Health Service
44. Are patients and families routinely advised on how to obtain information about the following? (Select all that apply)	☐ Accessing NDSS<br>☐ Health care card means tested Diabetes allowance<br>☐ Medicare<br>☐ Government subsidies - means tested Diabetes allowance<br>☐ Government subsidies - means tested carers allowance<br>☐ Government subsidies - essential medical equipment payment<br>☐ Housing allowance<br>☐ University entrance exam special conditions<br>☐ Driver's license sign-off<br>☐ Other (please specify below)
44a. Other (please specify)	__________________________________
45. Are children and young people offered information and contact details of local T1D support groups?	◯ Yes<br>◯ No<br>◯ Don't know
45a. Why not?	__________________________________
45b. Are the support groups accessible:	☐ Online or via social media<br>☐ In person<br>☐ Both<br>☐ Don't know
46. Does the service offer children and young people with T1D and their families access to mobile health information technology from the diabetes care team?	◯ Yes<br>◯ No<br>◯ Don't know
46a. Which ones? (select all that apply)	☐ Apps<br>☐ Text messages<br>☐ Other (please specify below)
46b. Which apps do you use?	__________________________________
46c. Why not?	__________________________________
46d. Other (please specify)	__________________________________

Ongoing diabetes care

47. Does your service offer any of the following education sessions or programs for Type 1 diabetes patients/families? Note this is not for initial diagnosis, but for ongoing care. (Select all that apply)

- ☐ Diabetes and exercise
- ☐ Diabetes and transition to adult services
- ☐ Diabetes and smoking
- ☐ Drugs and alcohol
- ☐ Diabetes and driving
- ☐ Diabetes and pregnancy
- ☐ Diabetes and Sick day management
- ☐ Diabetes technologies
- ☐ Insulin adjustment
- ☐ Nutrition and carbohydrate counting
- ☐ Pre-school to school transition preparation
- ☐ Preparation for Schoolies and independent travel
- ☐ No education sessions or programs are offered
- ☐ We use programs offered by other organisations e.g. Diabetes Australia
- ☐ Other (please specify)

47a. Other (please specify)

____

48. Do you regularly refer T1D patients to groups that offer patient education services, information and support?

- ◯ Yes
- ◯ No
- ◯ Don't know

48a. Which groups do you regularly refer patients to? (select all that apply)

- ☐ JDRF Australia or other JDRF state specific
- ☐ Diabetes Australia or other Diabetes Australia state specific
- ☐ Our own
- ☐ Online resources (not JDRF or Diabetes Australia)
- ☐ Other (please specify)

48b. Other (please specify)

____

49. Does your service have its own patient management resources for the following?

- ☐ Diabetes and exercise
- ☐ Diabetes and transition to adult services
- ☐ Diabetes and smoking
- ☐ Drugs and alcohol
- ☐ Diabetes and driving
- ☐ Diabetes and pregnancy
- ☐ Diabetes and Sick day management
- ☐ Diabetes technologies
- ☐ Insulin adjustment
- ☐ Nutrition and carbohydrate counting
- ☐ Pre-school to school transition preparation
- ☐ Preparation for Schoolies and independent travel
- ☐ No education sessions or programs are offered
- ☐ We use programs offered by other organisations e.g. Diabetes Australia
- ☐ Other (please specify)

49a. Would you be interested in sharing your resources with others?

- ◯ Yes
- ◯ No
- ◯ Don't know

49b. Other (please specify)

____

50. Would you be interested in having access to other services and resources for your patients?	◯ Yes<br>◯ No<br>◯ Don't know
50a. If you answered yes to either of the above questions, please provide your email.	__________________________________
51. Does your clinic undergo regular evaluations such as audits and accreditation?	◯ Yes<br>◯ No<br>◯ Don't know
51a. Would you be happy to share your evaluation report?	◯ Yes<br>◯ No<br>◯ Don't know
51b. If yes, please provide your email.	__________________________________
51c. Which health outcomes are reported and how often?	____
51d. Why not?	__________________________________

Insulin regimens

52. Does your service manage T1D patients with the following insulin regimens? (Select all that apply)

- ☐ 1-2 a day insulin injections
- ☐ 3 times a day insulin injections
- ☐ Multiple daily injections
- ☐ Continuous subcutaneous insulin infusion
- ☐ CGM and integration with hybrid closed loop pumps
- ☐ DIY pumps
- ☐ None of the above

52a. Do you have a service database documenting the insulin regimen for each patient?

- ◯ Yes
- ◯ No
- ◯ Don't know

53. Is there a trained specialist team available to initiate insulin pump therapy?

- ◯ Yes
- ◯ No
- ◯ Don't know

53a. Why not?

____

57. What key clinical outcomes do you routinely measure and record during consultations? (Select all that apply)

- ☐ Weight, height
- ☐ Blood pressure (systolic/diastolic)
- ☐ BGL readings
- ☐ HbA1c
- ☐ CGM glycaemic metrics eg %hypoglycaemia/ TIR/ % high, %V high, CV, other
- ☐ Pump settings and TDD
- ☐ Complications assessment
- ☐ None of the above
- ☐ Other (please specify below)

57a. Other (please specify)

____

58. Do you routinely download the data from patient's pumps or CGMs?

- ◯ Yes
- ◯ No
- ◯ Don't know

58a. Do you use the downloaded data in your clinical decision making?

- ◯ Yes
- ◯ No
- ◯ Don't know

58b. Why not?

____

59. Do you have standard ISPAD and NICE guideline congruent responses provided regularly to your patients on how to respond to out-of-range readings from pumps/glucometers/CGM regarding: (Select all that apply)

- ☐ Highs on sick days
- ☐ Regular patterns of high BGLs
- ☐ Hypoglycaemia on sick days
- ☐ Regular patterns of hypoglycaemia
- ☐ Ejection from auto mode
- ☐ Pump failure disconnection doses
- ☐ Screening for eating disorders of DE
- ☐ Screening for T1D complications
- ☐ Management for exercise
- ☐ Other (please specify below)

59a. Other (please specify)

____

Monitoring glycaemic control

60. What target HbA1c ranges does your service aim for in patients aged: 0-5 years? __________________________________

61. What target HbA1c, ranges does your service aim for in patients aged: 6-11 years? (mmol/ml) __________________________________

62. What target HbA1c ranges does your service aim for in patients aged: 12-18 years? (mmol/ml) __________________________________

63. What target HbA1c ranges does your service aim for in patients aged: 18 years and over? (mmol/ml) __________________________________

Optimal time in range

64. Do you have a clinic target for the optimal time in range for CGM across a week in patients aged: 0-5 years? __________________________________

65. What is the optimal time in range for CGM across a week in patients aged: 6-11 years in your clinic? __________________________________

66. What is the optimal time in range for CGM across a week in patients aged: 12-18 years in your clinic? __________________________________

67. What is the optimal time in range for CGM across a week in patients aged: 18+ years in your clinic? __________________________________

Hypoglycaemia targets

68. What is the target % Hypoglycaemia < 3.9 mmol/l on CGM in patients aged: 0-5 years in your clinic? __________________________________

69. What is the target % Hypoglycaemia < 3.9 mmol/l on CGM in patients aged: 6-11 years in your clinic? __________________________________

70. What is the target % Hypoglycaemia < 3.9 mmol/l on CGM in patients aged: 12-18 years in your clinic? __________________________________

71. What is the target % Hypoglycaemia < 3.9 mmol/l on CGM in patients aged: 18+ years in your clinic? __________________________________

72. Does your service include specific age-related glycaemic targets in the diabetes care plans and share them with patients/ families and schools?
- ◯ Yes
- ◯ No
- ◯ Don't know

72a. Why not? __________________________________

73. Does your service have data on how many patients reach target levels (i.e., HbA1c)?
- ◯ Yes
- ◯ No
- ◯ Don't know

73a. Why not? __________________________________

74. For patients not on CGM, how often does the service recommend children and young people test their blood glucose level?

	Testing for twice a day injections	Testing for three times a day injections	Testing for multiple daily injections	Testing for continuous subcutaneous insulin infusion
2-3 times	☐	☐	☐	☐
3-4 times	☐	☐	☐	☐
>4 times	☐	☐	☐	☐

78. What CGM device(s) is being used?

____

79. Are patients given education/instructions on how to check and calibrate their CGM?

◯ Yes
◯ No

79a. Is this done via? (Select all that apply)

☐ DE education session
☐ Patients do it themselves online
☐ Explanation during routine consultations
☐ Provided with written materials / guides
☐ Directed to a web resource
☐ Other (please specify below)

79b. Other (please specify)

____

80. Are children and young people with HbA1c levels consistently above 9.5% offered additional support?

◯ Yes
◯ No
◯ Don't know

80a. What sort of support? (Select all that apply)

☐ More frequent visits
☐ Telehealth with bloods
☐ Additional social work or psychologist support
☐ Ask patient to add weekly or 2 weekly CGM and pump uploads
☐ Suggest going to a Diabetes Australia camp (for children only)
☐ Admission for stabilisation
☐ Other (please specify below)

80b. Other (please specify)

____

81. Does the service follow the Diabetes Australia guidelines for optimal targets for short-term glycaemic control (pre-prandial 4 - 8mmol/litre and less than 10 mmol/litre 2 hours post prandial) for patients aged:
(Select all that apply)

☐ 0-5 years
☐ 6-11 years
☐ 12 years and over
☐ 18 years and over

82. On average, what percentage of your T1D patients have HbA1c levels ≤ 7.0?

- ◯ 0-10%
- ◯ 10-20%
- ◯ 20-30%
- ◯ 30-40%
- ◯ 40-50%
- ◯ 50-60%
- ◯ 60-70%
- ◯ 70-80%
- ◯ 80-90%
- ◯ 90-100%

83. On average, what percentage of your T1D patients are on twice a day (BD) insulin delivery?

- ◯ 0-10%
- ◯ 10-20%
- ◯ 20-30%
- ◯ 30-40%
- ◯ 40-50%
- ◯ 50-60%
- ◯ 60-70%
- ◯ 70-80%
- ◯ 80-90%
- ◯ 90-100%

84. On average, what percentage of your T1D patients are on three times a day (TDS) insulin delivery?

- ◯ 0-10%
- ◯ 10-20%
- ◯ 20-30%
- ◯ 30-40%
- ◯ 40-50%
- ◯ 50-60%
- ◯ 60-70%
- ◯ 70-80%
- ◯ 80-90%
- ◯ 90-100%

85. On average, what percentage of your T1D patients are on multiple daily injections or pump therapy?

- ◯ 0-10%
- ◯ 10-20%
- ◯ 20-30%
- ◯ 30-40%
- ◯ 40-50%
- ◯ 50-60%
- ◯ 60-70%
- ◯ 70-80%
- ◯ 80-90%
- ◯ 90-100%

86. One average, what percentage of your patients are on automated insulin delivery?

- ◯ 0-10%
- ◯ 10-20%
- ◯ 20-30%
- ◯ 30-40%
- ◯ 40-50%
- ◯ 50-60%
- ◯ 60-70%
- ◯ 70-80%
- ◯ 80-90%
- ◯ 90-100%

87. On average, what percentage of your T1D patients have < 4mmol/l hypoglycaemia?

- ◯ 0-10%
- ◯ 10-20%
- ◯ 20-30%
- ◯ 30-40%
- ◯ 40-50%
- ◯ 50-60%
- ◯ 60-70%
- ◯ 70-80%
- ◯ 80-90%
- ◯ 90-100%

88. On average what percentage of your T1D patients have TIR >70%?

- ◯ 0-10%
- ◯ 10-20%
- ◯ 20-30%
- ◯ 30-40%
- ◯ 40-50%
- ◯ 50-60%
- ◯ 60-70%
- ◯ 70-80%
- ◯ 80-90%
- ◯ 90-100%

89. If an audit has taken place during the past 12 months, on average, what percentage of your T1D patients get screened for complications?

- ◯ 0-10%
- ◯ 10-20%
- ◯ 20-30%
- ◯ 30-40%
- ◯ 40-50%
- ◯ 50-60%
- ◯ 60-70%
- ◯ 70-80%
- ◯ 80-90%
- ◯ 90-100%
- ◯ No audit has been taken

90. After exclusion of coeliac disease, thyroid disorder, Addison's disease and eating disorder, do children with T1D with recurrent seizures associated with low BGLs have access to the following: (Select all that apply)

- ☐ Neuropsychological assessments
- ☐ Screening for other disorders including psychological disorders
- ☐ No, but I would refer if the service was available
- ☐ No

Transition from paediatric to adult services

91. At what age do you assess readiness for transition to adult services?

____

92. At what age does your service require the patient to have transitioned to an adult service?

____

93. If the patient is not ready to transition by the prescribed age, will the transition occur anyway?

- ◯ Yes
- ◯ No
- ◯ Don't know

93a. Why?

____

94. Do you have any patients over 18 years in your service?

- ◯ Yes
- ◯ No
- ◯ Don't know

95. Do you have a specific transition service/ program or transition pathway available to your patients?

- ◯ Yes
- ◯ No
- ◯ Don't know

95a. Does your transition service include the following components: (Select all that apply)

- ☐ Transfer referral letter to adult service only
- ☐ Nurses in adult service meet patient before transfer
- ☐ Transfer to GP care only
- ☐ Structured education with acknowledgement of important knowledge bases before transition occurs
- ☐ Structured follow-up for assessment and confirmation of retainment in the adult service
- ☐ A young adult clinic
- ☐ Joint clinics where the paediatric team and the adult team will see the patient at the same time
- ☐ Group based, where a number of young people transition together as a cohort
- ☐ It is a virtual program
- ☐ Involves the family or other caregivers
- ☐ Provides specific preparation and education for family or other caregivers
- ☐ Case coordinator for each patient
- ☐ Does the paediatric service make the first appointment for the adult service
- ☐ A peer-mentor (or transition buddy) for each patient
- ☐ Other components (please describe below)

95b. Other components (please describe)

____

Effect of the pandemic and natural disasters

96. What issues do you foresee for your patients and your clinic now that we are living with COVID-19?

____

97. How have COVID-19 and the recent fires and floods influenced telehealth, telephone and face-to-face appointments in your area?

____

98. Please provide any other comments about your Type 1 Diabetes service here:

____

Type 2 Diabetes

99. Does your service look after children and young people with T2D?	◯ Yes<br>◯ No<br>◯ Don't know
99a. Are patients with T2D seen in a separate T2D clinic at a designated time?	◯ Yes<br>◯ No<br>◯ Don't know
99b. Do you see patients with T2D remotely (i.e., via outreach)?	◯ Yes<br>◯ No<br>◯ Don't know
99c. How many patients with T2D does your service care for per annum?	__________________________________
99d. Do you have specific T2D resources for your paediatric patients?	◯ Yes<br>◯ No<br>◯ Don't know
99e. What percentage of these T2D patients do you provide full diabetes care to?	◯ 0-25%<br>◯ 26-50%<br>◯ 51-75%<br>◯ 76%-100%
99f. Does your service provide expert advice to another healthcare professional or service for the ongoing care of patients with T2D?	◯ Yes<br>◯ No<br>◯ Don't know

Final questions

100. Do you provide data about your service to ADDN?	◯ Yes<br>◯ No<br>◯ Don't know
100a. Would you like to be contacted by someone from ADDN?	◯ Yes<br>◯ No
101. Would you be interested in contributing to a manuscript based on this survey? This would involve critically reviewing manuscripts.	◯ Yes<br>◯ No
102. Would you like it to be acknowledged by name as a contributor to the survey?	◯ Yes<br>◯ No
103. Would your service be happy to be contacted for any further surveys related to paediatric or transitional diabetes care?	◯ Yes<br>◯ No
104. Are there any services in your network that you would recommend taking this survey? If so, please provide their name and/or contact information.	______________________________
105. Do you have any other feedback about the survey?	________________________
106. Would you be willing to be contacted for a 45-minute interview about your service? This would enable us to gain a more in-depth understanding of how your service works and to use that information to help propose new service models	◯ Yes<br>◯ No
