# Supplementary material for: Health Service Leaders’ Perspectives on Type 1 Diabetes Models of Care for Children and Young Adults in Australia: A Mixed‐Methods Study
Source: J Diabetes Res. 2026 Apr 29;2026:7441677. doi: 10.1155/jdr/7441677 (PMC13128981; doi:10.1155/jdr/7441677)
Supplement: Supplementary file 2 — Supporting Information 2 Supplemental Documentation Table S1: Provider role, service location and setting (n = 7). [file JDR-2026-7441677-s002.docx]

**Supplemental Documentation Table 1**

**Supplemental Documentation Table 1. Provider role, service location and setting (n=7)**

| **Provider** | **Role** | **State** | **Metropolitan/**  **regional/rural** | **Service setting** |
| --- | --- | --- | --- | --- |
| 1 | Paediatric endocrinologist | Tasmania | Metropolitan | Paediatric outpatient clinic (adult hospital) |
| 2 | Diabetes educator | Tasmania | Metropolitan | Paediatric outpatient clinic (adult hospital) |
| 3 | Diabetes educator | Tasmania | Metropolitan | Paediatric outpatient clinic (adult hospital) |
| 4 | Paediatric endocrinologist | Western Australia | Metropolitan but does outreach | Paediatric outpatient clinic (children’s hospital) |
| 5 | Paediatric endocrinologist | Queensland | Regional | Paediatric outpatient clinic (adult hospital) |
| 6 | Paediatric endocrinologist | New South Wales | Metropolitan | Paediatric outpatient clinic (children’s hospital) |
| 7 | Clinical Nurse Consultant | New South Wales | Regional | Paediatric outpatient clinic in an adult hospital |
