# Supplementary material for: Health Service Leaders’ Perspectives on Type 1 Diabetes Models of Care for Children and Young Adults in Australia: A Mixed‐Methods Study
Source: J Diabetes Res. 2026 Apr 29;2026:7441677. doi: 10.1155/jdr/7441677 (PMC13128981; doi:10.1155/jdr/7441677)
Supplement: Supplementary file 3 — Supporting Information 3 Table S2: Thematic analysis summary. [file JDR-2026-7441677-s003.docx]

**Supplemental Documentation Table 2.** Thematic analysis summary

**Table 2**. Thematic analysis summary

| **Theme** | **Sub-theme** | | | **Description** | **Illustrative quotes** | |
| --- | --- | --- | --- | --- | --- | --- |
| ***Perspectives on what is working well in the service*** | | | | | | |
| **Teams and staff** | 1. Skilled staff and MDT  (n=5) | | | Collaborative, skilled MDT with diabetes knowledge that was patient and family focused. | *A motivated team that maintains focus on patient and family-centred care. The focus is on improving outcomes. Nothing specific or measurable, just providing children with a good quality of life. (Provider 5)*  *They teach the parents/children about responsibility from their diagnosis. What child can do, capacity building. (Provider 1)* | |
|  | 2. Consistent care  (n=4) | | | Dedicated, stable, and cohesive teams building relationships with patients and caregivers. Ensures that families were seen by the same providers (e.g., paediatrician and diabetes educator) when possible. | *Patients tend to stay, so there is a type of ownership. (Provider 2)*  *Low turnover of staff maintains consistency [for patients]. … see same doctor and services. (Provider 7)* | |
|  | 3. Ability to provide support  (n=4) | | | High level of staff resources and service agreements.  Outreach and equitable care for patients living in remote areas.  Ability to provide access to out-of-hours care. | *[We are] well stocked with senior and junior physicians, diabetes educators, and dieticians. (Provider 6)*  *Support to take multidisciplinary teams outside of metropolitan areas to outer clinics [who are protected by service agreements]. (Provider 4)*  *Provision of 24x7 service ensuring quick access to doctors and nurses [for both inpatient and outpatient care]. Dieticians, nurses, and social workers are embedded in outpatient care across all clinics. (Provider 4)* | |
|  | 4. Processes  (n=1) | | | Regular quarterly appointments for all children; each child has their own clinic group. | *[Our state] follows a public model of diabetic care wherein each child has their own clinic group with appointments mapped 12 months in advance, ensuring equitable service delivery. (Provider 4)* | |
| **Data infrastructure and tools** | 5. Communication  (n=4) | | | Digital communication tools enabled patient access to providers via SMS texts and emails. Ability to see a patient’s CGM data facilitated faster responses to issues. | *Communication with families, texts, emails, they look at patient’s CGM; patients are open about the data. (Provider 3)* | |
|  | 6. Processes  (n=1) | | | Reporting of baseline clinic data  on service performance.        Adequate resources to support education on technology. | [*We have*] *reasonable quality data on outcomes (and infrastructure that supports this). We can collect baseline data on service performance to track over time and measure influence on outcomes. (Provider 4)*    *Dedicated time to develop technological programs for youth. Also, dedicated FTE [full-time equivalent] for nurses to deliver education and technology programs for youth. (Provider 4)* | |
| ***Perspectives on challenges*** | | | | | | |
| **Coordinated care across health providers** | | *1. Disconnection between T1D MDTs and primary healthcare (n=6)* | Need for greater GP integration with specialist MDT team. Limited patient access to GPs means increased MDT clinic workload about non-diabetes concerns raised by the patients/families. | | | *As soon as diagnosed, families bypass a GP and expect to get everything at the tertiary center. We get a lot of stuff that should be managed in primary and secondary care but is using tertiary resources. (Provider 6)*    *Once discharged, [patients] can’t access social work, and they need to go back to GP for a [mental health] plan. For kids we don’t do complication screening. [There is a] disconnect as we have to send out externally. (Provider 7)* |
| **Composition of teams and staff availability** | | 2. Mental health staff shortages (n=6) | Mental health staff shortages specifically psychologists, counsellors, social workers,  and mental health workers. | | | *[We are] under-resourced for social workers, who are having to do psychologists’ work. Don’t have psychologists. Issue with diabetes burn out, especially for families that are struggling. (Provider 6)*    *We need increased psychologists’ hours. For example, we can’t screen for body dysmorphia even though we suspect an issue. (Provider 2)*    *The service becomes disjointed as they have to send back to GP for a mental health plan to see a psychologist in the community who may not have any diabetes-specific knowledge. (Provider 6)*    *Early proactive mental health support for families starting from early detection stages[is needed].(Provider 4)* |
|  |  | 3. Clinical staff shortages  (n=4) | A lack of funding for appropriate staff types limits ability to liaise with schools, creates barrier to staff training, and hinders provision of person-centred integrated care. | | | *No funding for dietician at this time. Funding is not secured for psychologist, social worker, staff to visit schools. Only just meet KPI [key performance indicator] in schools; in all other areas don’t. (Provider 2)*    *[We need] enough staff to support service and funding. To dedicate funds to staff so they can do what they are meant to do, support schools, CPD [continuing professional development], conferences. (Provider 1)*    *Specifically, someone in a coordinator role in the clinic to manage appointments, book follow-ups, etc. (Provider 5)*    *Limited ability to provide flexible care. Most kids need more individualised appointments than four times a year, but we are unable to add them in. (Provider 2)* |
|  |  | 4. Recruitment challenges  (n=2) | Part-time and shared staff creates concern about access to care.  Challenges with funding and staff recruitment. | | | *We put in applications for transition funds and hours for staffing and are never successful as no money. Give up as never successful. (Provider 7)*    *The diabetes educator is shared with the adult clinic [as the] priority is adults. Constant worry from allied health services due to stretched workload. (Provider 5)* |
| **Inequitable access** | | 5. Challenges in rural regions  (n=3) | Differential resourcing. Care continuity | | | *Compared with ISPAD recommendations, need to be careful in setting the benchmark to clinics in the city. They are also under-resourced so it would not be fair to look at them and decide what the regional clinic needs. For example, they might not have a psychologist on board but that does not mean that regional clinic does not need one. (Provider 5)*    *Key challenges are for youth who need to travel to metropolitan areas for treatment. They also might not come to the same place across 12 months so harder to manage their appointments. Provider 4*)*,* |
|  | | 6. Inequitable funding models  (n=1) | Inequitable access to diabetes technologies for families that do not have private health insurance. | | | *Most funding comes from silver-tier private insurance, but only 30–40% people have this coverage.* (*Provider 6*) |
